# Medication errors as malpractice-a qualitative content analysis of 585 medication errors by nurses in Sweden

**DOI:** 10.1186/s12913-016-1695-9

**Published:** 2016-08-24

**Authors:** Karin Sparring Björkstén, Monica Bergqvist, Eva Andersén-Karlsson, Lina Benson, Johanna Ulfvarson

**Affiliations:** 1 Department of Neurobiology, Care Sciences and Society, Karolinska Institutet, Stockholm, Sweden; 2 Department of Clinical Science and Education Södersjukhuset, Karolinska Institutet, Stockholm, Sweden; 3 Department of Internal Medicine, Södersjukhuset AB, SE-118 83 Stockholm, Sweden; 4 Psychiatry South Stockhholm, Administration och Ledning, Box 5040, SE-121 05 Johanneshov, Sweden

**Keywords:** Medication error, Classification, Contributory factor, Nurse, Malpractice

## Abstract

**Background:**

Many studies address the prevalence of medication errors but few address medication errors serious enough to be regarded as malpractice. Other studies have analyzed the individual and system contributory factor leading to a medication error. Nurses have a key role in medication administration, and there are contradictory reports on the nurses’ work experience in relation to the risk and type for medication errors.

**Methods:**

All medication errors where a nurse was held responsible for malpractice (*n =* 585) during 11 years in Sweden were included. A qualitative content analysis and classification according to the type and the individual and system contributory factors was made. In order to test for possible differences between nurses’ work experience and associations within and between the errors and contributory factors, Fisher’s exact test was used, and Cohen’s kappa (k) was performed to estimate the magnitude and direction of the associations.

**Results:**

There were a total of 613 medication errors in the 585 cases, the most common being “Wrong dose” (41 %), “Wrong patient” (13 %) and “Omission of drug” (12 %). In 95 % of the cases, an average of 1.4 individual contributory factors was found; the most common being “Negligence, forgetfulness or lack of attentiveness” (68 %), “Proper protocol not followed” (25 %), “Lack of knowledge” (13 %) and “Practice beyond scope” (12 %). In 78 % of the cases, an average of 1.7 system contributory factors was found; the most common being “Role overload” (36 %), “Unclear communication or orders” (30 %) and “Lack of adequate access to guidelines or unclear organisational routines” (30 %). The errors “Wrong patient due to mix-up of patients” and “Wrong route” and the contributory factors “Lack of knowledge” and “Negligence, forgetfulness or lack of attentiveness” were more common in less experienced nurses. The experienced nurses were more prone to “Practice beyond scope of practice” and to make errors in spite of “Lack of adequate access to guidelines or unclear organisational routines”.

**Conclusions:**

Medication errors regarded as malpractice in Sweden were of the same character as medication errors worldwide. A complex interplay between individual and system factors often contributed to the errors.

## Background

Medication errors (MEs) are probably the most common type of patient safety incidents worldwide and cause harm to patients, distress to medical staff and costs to the health care system. Systematic reporting of errors is fundamental for detecting patient safety problems, but there is no consensus neither concerning the terminology of MEs nor the error reporting systems. A few countries have developed advanced national incident reporting systems but a variety of local reporting systems are used elsewhere [1]. The International Medication Safety Network (IMSN) aims to develop a common and systematic terminology as a basis for powerful prevention strategies [2].

The five most common methods for detecting MEs are: 1) studies of errors reported to the authorities, 2) studies of errors reported spontaneously to local reporting systems, 3) screening of medication orders and patient medical charts, 4) observational studies, and 5) qualitative studies in which healthcare personnel are interviewed.

Probably, only about 5 % of MEs are reported [3, 4]. The traditional roles of reporting systems are punitive. Health care professionals may be criticized or even lose their licence. Reporting an error is often regarded as a way to blame and shame, although MEs often arise due to the complex interplay between circumstances in a busy clinical setting [5]. A non-punitive ME reporting system, however, can be used to learn from errors, provide feedback to those involved and improve routines.

The lack of a generally accepted terminology makes comparisons difficult. A recent meta-analysis from the United Kingdom revealed three different definitions of medication administration errors, 44 sub-categories and four denominators [6]. Some researchers have suggested how to analyse and classify the underlying reasons that lead to MEs as reviewed by Brady et al. [7]. Insignificant MEs are more common than hazardous MEs and may or may not have different reasons and underlying mechanisms [8, 9].

Registered nurses (RNs) are responsible for medication administration in most setting and are therefore the health professionals most often reported for MEs [10]. Depending on workplace, RNs spend up to 40 % of working hours administering and managing drugs [11], and are the gate-keepers for intercepting MEs and mitigating its consequences [12].

The clinical experience of the nurse is important to what kind of unsafe actions the nurse may take before an error occurs [13]. Clinically inexperienced nurses may be more affected by system factors than experienced nurses. Studies linking nurses’ clinical experince and MEs are, however, inconclusive. Some studies have shown correlations between inexperienced nurses and an increased risk for MEs [14–18], whereas others did not find such correlations [19, 20].

At the time of the study, all Swedish healthcare providers were required to report potentially dangerous treatment-related adverse events to the National Board of Health and Welfare (NBHW). The NBHW could criticize individual health care professionals as well as the routines in the health-care unit. The crucial point was not the actual harm to the patient but the potential risk of an event. In rare cases, the NBHW could report individuals to a criminal court, but Sweden does not have a tradition of taking medical issues to court. The system has gradually been replaced by local reporting systems intended to detect system errors rather than criticizing individuals, but the most serious cases must today be reported to a new authority, the Health and Social Care Inspectorate (IVO).

There are many studies that survey the number and the type of reported MEs in different settings, but few in-depth studies focussing on hazardous MEs made by nurses. In order to get a deeper understanding of the process leading a hazardous ME, we conducted a two-phase study of ME by nurses serious enough to be judged as malpractice in Sweden. The first phase served to develop a classification system for MEs and system and individual contributory factors based on 33 medication errors [5]. The present paper reports the second phase where all MEs during 11 years where a nurse was found responsible by the NBHW were analyzed using the above classification system.

The aim of this work was to increase the understanding of potentially hazardous MEs by nurses with regard to the type of error, the individual contributory factors and the work experience of the nurse and the system contributory factors of the workplace.

## Methods

Cases were selected from the NBHW reports of potentially hazardous reported MEs in Sweden. All reported MEs from Jan 1, 1996 to Dec 31, 2006 where a nurse was held responsible for malpractice were selected (*n =* 585). The case files included the original report about the error, the patient records, event descriptions from the nurse and other medical staff involved and sometimes from the patients and finally the assessment and conclusions by the NBHW. Some case files were short and uncomplicated whereas others were extensive. The complete case files were photo-copied and formed the base for the study, and were then systematically scrutinized.

The 585 MEs were made in a variety of health care settings; 243 cases (42 %) in hospital based care, 221 cases (38 %) in nursing homes, 63 cases (11 %) in home care services and 44 cases (8 %) in outpatient care. Health care setting was missing in 14 (2 %) cases.

The patients were aged 2 months−98 years of age. There were 43 children <18 years of age (7 %), 156 patients aged 18–65 years of age (27 %) and 354 patients >65 years of age (60 %). Patient age was missing in 32 cases (6 %). There were 310 female (53 %) and 248 male (42 %) patients. Patient gender was missing in 32 cases (6 %).

All the nurses were registered nurses (RNs). Sweden does not have a system for grading nurses. Mean age was 44 (Range 24–64) years of age. There were 444 women (76 %) and 79 men (13 %). Gender was missing in 62 cases (11 %). There were 317 nurses with >2 years of work experience (54 %) and 55 nurses with <2 years of work experience (9 %). Work experience was missing in 213 of the cases (36 %). Mean work experience was 10 years.

A qualitative content analysis was conducted on the narratives in the 585 case files to classify the errors into different types and to identify the individual contributory factors (ICFs) and the system contributory factors (SCFs) that preceded the error using the analysis procedure described by Graneheim and Lundman [21].

Three RN researchers trained in qualitative methodology made the content analysis and classified the cases according to the type of ME and contributing factors using a modification of the classification described by Bergqvist et al. [5].

The analysis was made in four steps. Firstly, the case file was read several times to get an understanding of the whole. Secondly, the text was divided into meaning units. Thirdly, the meaning units were summarized (condensed) and labelled with codes. Finally, the codes were sorted into subcategories and main categories which were based on the comparison on the similarities and differences (Graneheim and Lundman, 2004). The four-step content analysis process is demonstrated in Table 1.

**Table 1:** The four-step content analysis process

Meaning unit	Condensation		Main category	Sub-category
Nurse finds an opened vial with sodium chloride solution on the tray “as it usually is” and injects 5 ml. Later, two unopened vials of sodium chloride solution and one opened vial of potassium chloride solution are found on the tray.	Nurse found an opened vial of sodium chloride. “As it usually is.” “No thought that it could be anything else”	Potassium chloride instead of sodium chloride	Medication error type	Wrong drug due to mix-up of drugs
“I read on the vial, but I didn’t notice what it said.” “She didn’t notice of the small dose prescribed, but took it for granted that the patient should have an ordinary dose.” “I totally forgot about the medication.”	Did not pay attention to the content of the text Didn’t notice and took for granted Forgot to give the medicine to the patient	Insufficient attention Took for granted Forgot	Individual factor	Negligence, forgetfulness or lack of attentiveness
“I was alone and the medication administration must be done at all the wards at the same time”	Alone with all administration at the same time	To much work to perform in insufficient time	System factor	Role overload

Table 1 shows examples of the four-step content analysis. After careful reading, the text was divided into meaning units, and then condensed. The codes were sorted into subcategories and main categories

The MEs were assigned to nine categories as shown in Table 2: 1) wrong dose, 2) wrong drug due to mix-up of drugs, 3) wrong patient due to mix-up of patients, 4) Omission 5) unauthorized drug, 6) wrong route, 7) wrong judgement or inadequate assessment of the patient’s need for treatment, 8) wrong management or storage of the drug, and 9) allergy-related error. The categories “Wrong patient”, “Wrong judgement (or inadequate assessment of the patient’s need for treatment)” and “Wrong management or storage of the drug” were, not present in the methodological study built on a smaller material (*n =* 33), but were developed for this study by the same researchers using the same method [5].

**Table 2:** The nine different categories of medication errors

Error type	*N* (%)	Stories illustrating the categories
Wrong dose	241 (41)	*Due to bad communication between a nurse and an assistant nurse, they both gave a woman with diabetes insulin.*
Wrong drug	96 (16)	*A man with heart failure was given morphine instead of furosemide intravenously because the nurse was thinking of another patient.*
Wrong patient	76 (13)	*An in-patient was given drugs that were meant for the patient in the next bed.*
Omission	69 (12)	*A patient did not get warfarin for 2 weeks since the medication was temporarily discontinued and then forgotten.*
Unauthorized drug	57 (10)	*A nurse gave a patient with severe pain a higher dose of analgesics than prescribed because the lower dose had not resulted in pain relief. No physician was available at the time.*
Wrong route	35 (6)	*Due to a misunderstanding of the nurse’s instructions, an assistant nurse administered ear drops into the eyes of a nursing home patient.*
Wrong judgement (or inadequate assessment of the patient’s need for treatment)	16 (3)	*A patient who had very a low blood sugar was nevertheless given her prescribed dose of insulin.*
Wrong management or storage of the drug,	11 (2)	*A nurse who could not find the proper drug picked up a package from a box of discarded drugs and gave it to the patient.*
Allergy-related error	9 (2)	*Using the department’s the list of drugs that nurses are allowed to administer occasionally without a doctor’s order, a nurse administered alimemazine to a patient from without noticing that the patient was allergic to this drug.*
Other	3 (<1)	
Total numbers of errors in the 585 cases	613	

Table 2 shows the nine different categories of medication errors, the number and the percent calculated in relation to the 585 cases for each category and examples illustrating each category

The ICFs were assigned to six categories as shown in Table 3: 1) negligence, forgetfulness or lack of attentiveness, 2) proper protocol not followed, 3) lack of knowledge, 4) practice beyond scope of practice, 5) inappropriate communication (including documentation errors), and 6) disease or drug abuse. The SCFs were assigned to eight categories as shown in Table 4: 1) role overload, 2) unclear communication or orders, 3) lack of adequate access to guidelines or unclear organisational routines, 4) inappropriate location of medication or look-alike medication, 5) interruption or distraction when preparing or administering medication, 6) inadequate technique or pharmaceutical service, 7) pressure from patient/patient’s family or other staff members to satisfy the patient’s immediate need, and 8) administration in an emergency situation. The categories” Inadequate technique or pharmaceutical service” and “Administration in an emergency situation” and “Unclear communication or orders” were not present in the methodological study but were developed for this study by the same researchers using the same method [5]. The categories “organizational routines” and “lack of adequate access to guidelines or information” were due to overlap treated as one category “Lack of adequate access to guidelines or unclear organizational routines”.

**Table 3:** The six different categories of individual contributory factors

Individual contributory factors	*N* (%)	Stories illustrating the categories.
Negligence, forgetfulness or lack of attentiveness	399 (68)	*One nurse put two vials on the table in a cancer patient’s home. The prescription was Hydromorphone 10 mg/ml, 1.8 ml s.c. and Morphine 10 mg/ml, 9 ml i.v. Hydromorphone is five times stronger than Morphine.* *Another nurse gave an intravenous injection from a 10 ml. vial. When she was about to give Hydromorphone, that she believed existed only in 1 ml vials, she could not find it on the table. She now realized that she has given Hydromorphone instead of Morphine.* *A temporarily employed doctor had prescribed ordered both Morphine and Hydromorphone in 10 ml vials.*
Proper protocol not followed	147 (25)	*A man with acute stroke had recently been treated for a myocardial infarction and was therefore admitted to the cardiology department for treatment of his stroke. He was prescribed ateplase as thrombolytic therapy. The nurse was familiar with this drug in cardiology practice but not for stroke.* *Alteplase consists of two vials to be mixed by the nurse, but the dosage and the preparation are different for different diagnoses. With one of the vials in her hand, the nurse walked around and asked several doctors and nurses about the procedure. Then she mixed the ateplase infusion and administered it to the patient.* *The next morning, the vial with the active ingredient was found at the table in the doctor’s office.*
Lack of knowledge	76 (13)	*The same case as Proper protocol not followed.*
Practice beyond scope of practice	68 (12)	*A nursing home patient with severe cancer pain was prescribed dextropropoxyphene and paracetamol 4 times a day and morphine 5 mg “when needed”.* *On a Saturday, pain had worsened in spite of morphine 4 doses daily. The RN changes the order to paracetamol and morphine 5 mg four times daily and excluded dextropropoxphene.* *The physician thought that the RN had passed her authorization when not consulting a physician.*
Inappropriate communication	62 (11)	*A nursing home patient was prescribed tramadol 1–2 tablets “when needed”. The RN put 8 tablets in a medicine cup labelled* “*tramadol when needed”.* *An assistant nurse with an authorization to administer drugs gave the patient all 8 tablets at the same time.* *The RN had failed in her communication to the assistant nurse giving incomplete information.*
Disease or drug abuse	203 (3)	*The nurse had used the patient’s morphine herself.* *The patient did not get any morphine.*
No individual factor identified	29 (5)	
Total numbers of individual factors in the 585 cases	772	

Table 3 shows the six different categories of individual contributory factors, the number and the percent calculated in relation to the 585 cases for each category and examples illustrating each category

**Table 4:** The eight different categories of system contributory factors

System factors	*N* (%)	Stories illustrating the categories
Role overload	212 (36)	*A child with a heart failure in an intensive care unit was to be transported to another hospital. The nurse could not find sodium chloride solution to flush the peripheral intravenous catheter and went into another patient room where he found a tray of opened sodium chloride solution vials.* *He flushed the catheter and the child immediately turned blue and stopped breathing.* *Two unopened vials of sodium chloride and one opened vial of potassium chloride were found on the tray.* *The nurse had felt stressed. The child had been in a serious condition during the night, and was not fed: The papers were not ready and the transport car did not have access to oxygen.*
Inappropriate location of medication or look-alike medication	79 (14)	
Unclear communication or orders	177 (30)	*An 80-year old lady was transferred from the intensive care unit to a general ward.* *A nurse copied the patient’s drug orders from the intensive care list to the medical care list by hand.* *She wrote “Digoxin 0.25 mg. 1 + 1 + 1” instead of “Digoxin 1 + 0 + 0”. The physician signed the order without noticing the error.* *The patient got the higher dose during 12 days.*
Lack of adequate access to guidelines or unclear organisational routines “The case also illustrates “Proper protocol not followed” in Table III	176 (30)	*A man with acute stroke had recently been treated for a myocardial infarction and was therefore admitted to the cardiology department for treatment of his stroke. He was prescribed alteplase as thrombolytic therapy. The nurse was familiar with this drug in cardiology practice but not for stroke.* *Alteplase consists of two vials to be mixed by the nurse, but the dosage and the preparation are different for different diagnoses. With one of the vials in her hand, the nurse walked around and asked several doctors and nurses about the procedure. Then she mixed the ateplase infusion and administered it to the patient.* *The next morning, the vial with the active ingredient was found at the table in the doctor’s office.*
Interruption or distraction when preparing or administering medication	47 (8)	*A nurse was preparing an infusion of furosemide and sodium chloride and in the room for storage and preparation of medications.* *The door was open and another patient in pain asked for morphine and begged the nurse to hurry up. The nurse replied that she would first finish what she started and then come with the morphine. The patient was standing in the door and talked to the nurse when she prepared the infusion.* *She mixed morphine instead of furosemide in the infusion and gave to a patient with heart failure.* *The nurse wrote that when she prepared the infusion, she was thinking of the patient needing morphine.*
Inadequate technique or pharmaceutical service	31 (5)	*A patient would receive chemotherapy and the pharmacy delivered the wrong drug. The name of the mis-delivered vial was long and therefore an abbreviation was used. The difference in name between the various drugs did not appear in the abbreviation.* *The nurse read the abbreviation on the vial and compared with the prescription.* *The nurse took for granted that it was the right drug delivered and gave it to the patient.*
Pressure from patient/patient’s family or other staff members to satisfy the patient’s immediate need	28 (5)	*A father came with his 7 year old son to the health centre. He claimed that it was the day for his son to get his monthly injection of growth hormone.* *The RN had worked in the centre for 2 weeks and could not find any notes about the injection in the child’s medical record. She questioned if it was the right day and what dose to give. The father was stubborn and claimed that he knew the dose and that his son must have his injection.* *The father seemed trustworthy and the RN gave the boy the injection.* *The boy got a too high dose and one week too early.*
Administration in an emergency situation	7 (1)	*The patient got a double dose of furosemide due to a communication misunderstanding in an acute situation.*
None	130 (22)	
Total numbers of system factors in the 585 cases	757	

Table 4 shows the eight different categories of system contributory factors, the number and the percent calculated in relation to the 585 cases for each category and examples illustrating each category

Patient harm was classified as no harm, moderate harm, serious (permanent) harm, and death.

### Statistics

Number (*n*) and percent (%) were reported for the ME types, ICFs, and SCFs for all cases and, in addition, also split by nurses’ work experience. In order to test for possible differences between work experience and associations within and between the errors and contributory factors, Fisher’s exact test was used, and Cohen’s kappa (k) was performed to estimate the magnitude and direction of the associations. All analyses were performed in R, version 3.1.3 (R Foundation for Statistical Computing, Vienna, Austria).

## Results

### The medication errors (MEs)

A total of 613 MEs were found in the 585 case files. The most common ME was “Wrong dose” in 41 % of the cases. “Wrong dose” correlated negatively with “wrong drug” (*k* = −0.28, *p <*0.001), “wrong patient” (*k* = −0.25, *p <* 0.001) and “omission” (*k* = −0.21, *p <* 0.001). Other common MEs were “Wrong drug” (16 %), “Wrong patient” (13 %) and “Omission of drug” (12 %). In three cases, the information available was in-sufficient to determine the error type. Table 2 shows the frequency of the nine different error categories along with illustrating examples.

### The individual contributory factors (ICFs)

In 556 of the cases (95 %), one or more ICFs were found. The total number was 772, so there was an average of 1.4 ICFs per case when present. No specific individual factor could be identified in 29 cases (5 %). The most common ICF was “Negligence, forgetfulness or lack of attentiveness” in 68 % of the cases. “Proper protocol not followed” occurred in 25 % and “Lack of knowledge” in 13 % of the cases. “Practice beyond scope” occurred in 12 % of the cases and correlated strongly and positively with “unauthorized drug” (*k* = 0.65, *p <* 0.001) and negatively with “Negligence, forgetfulness or lack of attentiveness” (*k =* −0.22, *p <* 0.001). “Inappropriate communication” occurred in 11 % of the cases and correlated positively with “Omission” (*k* = 0.25, *p <* 0.001). Table 3 shows the frequency of the different ICFs along with stories illustrating the categories.

### The system contributory factors (SCFs)

In 455 of the cases (78 %), one or more SCFs were found. The total number was 757 so when present, there was an average of 1.7 SCF per case. No SCF could be identified in 130 cases (22 %). The most common SCF was “Role overload” (36 %) followed by “Unclear communication or orders” (30 %) and “Lack of adequate access to guidelines or unclear organisational routines” (30 %). “Inappropriate location of medication or look-alike medication” occurred in 14 % and correlated strongly and positively with “Wrong drug” (*k* = 0.52, *p <* 0.001). “Interruption or distraction when preparing or administering medication” occurred in 8 % of the cases. Table 4 shows the frequency of the eight SCFs along with illustrating examples.

### Experienced versus un-experienced nurses

Nurses with less than 2 years work experience were significantly more likely to make the error “Wrong patient due to mix-up of patients” (*p <* 0.007) and the error “Wrong route”. (*p <* 0.003) than their more experienced colleagues. The un-experienced nurses were also more prone to the ICFs “Lack of knowledge” (*p <* 0.001) and “Negligence, forgetfulness or lack of attentiveness” (*p <* 0.007) and the experienced nurses were more prone to “Practice beyond scope of practice” (*p <* 0.004) and to make MEs in spite of “Lack of adequate access to guidelines or unclear organisational routines” (*p <* 0.035). Table 5 shows the number and percent of the MEs, ICFs and the SCF split by nurse’s work experience, and the *p*-values from Fisher’s exact test.

**Table 5:** The medication errors, the individual contributory factors and the system contributory factors in relation to the nurses’ work experience

		Nurse’s work experience		
		0–2 years	>2 years	*p*
		*n =* 55	*N =* 317	
Error type				
1	Wrong dose	20 (36.4 %)	140 (44.2 %)	0.305
2	Wrong drug due to mix-up of drugs	9 (16.4 %)	51 (16.1 %)	1.000
**3**	**Wrong patient due to mix-up of patients**	**14 (25.5 %)**	34 (10.7 %)	**0.007**
4	Omission	3 (5.5 %)	35 (11.0 %)	0.332
5	Unauthorized drug	2 (3.6 %)	35 (11.0 %)	0.139
**6**	**Wrong route**	**9 (16.4 %)**	14 (4.4 %)	**0.003**
7	Wrong judgement or inadequate assessment of the patient’s need for treatment	1 (1.8 %)	9 (2.8 %)	1.000
8	Wrong management or storage of the drug	1 (1.8 %)	7 (2.2 %)	1.000
9	Allergy-related error	0 (0.0 %)	5 (1.6 %)	1.000
Individual contributory factor				
**1**	**Negligence, forgetfulness or lack of attentiveness**	**46 (83.6 %)**	207 (65.3 %)	**0.007**
2	Proper protocol not followed	13 (23.6 %)	82 (25.9 %)	0.867
**3**	**Lack of knowledge**	**17 (30.9 %)**	37 (11.7 %)	**0.001**
**4**	**Practice beyond scope of practice**	1 (1.8 %)	**48 (15.1 %)**	**0.004**
5	Inappropiate communication	3 (5.5 %)	33 (10.4 %)	0.328
6	Disease or drug abuse	1 (1.8 %)	13 (4.1 %)	0.703
System contributory factor				
1	Role overload	25 (45.5 %)	117 (36.9 %)	0.233
2	Unclear communication or orders	13 (23.6 %)	87 (27.4 %)	0.624
**3**	**Lack of adequate access to guidelines or unclear organisational routines**	9 (16.4 %)	**97 (30.6 %)**	**0.035**
4	Inappropriate location of medication or look-alike medication	7 (12.7 %)	40 (12.6 %)	1.000
5	Interruption or distraction when preparing or administering medication	8 (14.5 %)	22 (6.9 %)	0.064
6	Inadequate technique or pharmaceutical service	2 (3.6 %)	23 (7.3 %)	0.557
7	Pressure from patient/family or other staff members to satisfy the patient’s immediate needs	2 (3.6 %)	21 (6.6 %)	0.551
8	Administration in an emergency situation	1 (1.8 %)	6 (1.9 %)	1.000

Table 5 shows the number of medication errors, individual contributory factors and system contributory factors (*n* and %) split by the nurses’ work experience: 0–2 years or >2 years. and *p*-values from Fisher’s exact test. Bold data show the statistically significant correlations

### Patient outcome

In 9 cases (1.5 %), the patients died as a result of the ME. In 29 cases (5 %), the patient was seriously harmed, in 64 cases (11 %) moderately harmed, and 466 (80 %) not harmed at all. Data was missing in 17 cases.

## Discussion

The MEs and their contributing factors in this study of malpractice were mainly of the same types that were common in other studies around the world. We did not, however, find any cases of poor calculation skills, previously often blamed as an important cause of MEs [22, 23]. Today, nursing schools emphasize calculation skills and physicians must write exact orders. “Wrong dose” was overwhelmingly common (41 %) and was frequent in other studies like in US emergency departments (18 % out of 13,932 reported MEs) [10] and in the national reporting system of England and Wales (15.23 % out of 526 186 reported errors) [24]. Those studies, however, include ME by others than RNs. Detailed comparisons of prevalence are inappropriate due to the heterogeneity of definitions, health care facilities, and reporting systems.

Some MEs like “Wrong patient” are always wrong, whereas others are relative. Three unquestionable types of MEs in our material were more common than in England and Wales 2005–2010: “Wrong route” occurred in 6 % in our study but only in 1.51 % in the England and Wales study: “Wrong drug” 16 % compared to 9.28 %; “Wrong patient” 13 % compared to 4.16 % [24]. MEs of a type that is always wrong like “Wrong patient” are probably more likely to be reported and are more difficult to conceal. When a lot of consideration is needed to assess whether an ME is present or not, it is less likely to be reported. If a nurse does not give insulin to a patient who had not eaten much, it may be classified as “Omission” or as good judgement depending on what measures are taken to check the patient’s condition and needs. Inexperienced nurses were much more likely to make the ME “Wrong route” and “Wrong patient”. Since every senior health care professional personally has experienced mix-up of patients, they have learnt to always double-check patient identity. Experience is also helpful to understand the variety of medication routes.

In 95 % of the cases, one or more ICF were present and in 78 % one or more SCF showing the complexity of MEs. The most common ICF “Negligence, forgetfulness or lack of attentiveness” occurred in two thirds of the cases. The most frequent SCFs were “Role overload”, “Unclear communication or orders” and “Lack of adequate access to guidelines or unclear organisational routines”. Although distractions and interruptions are frequently blamed for errors in other studies [25, 26] the SCF “Interruption or distraction when preparing or administering medication” occurred only in 8 % of the cases in the present study. Our figure is similar to 7.5 % in US emergency departments [10], but in a psychiatric setting, distraction was cited as the most common contributory factor (15.2 %) [27]. We did not find that clinically inexperineced nurses were more affected by system factors than experienced nurses. “Wrong drug” correlated strongly with the SCF “Inappropriate location of medication or look-alike medication”. This is a system factor that easily can be improved.

Two thirds of the patients in this study were older than 65 or younger than 18 years of age. Most pharmaceutical studies exclude the youngest, the oldest and the most vulnerable patients. In the 21st century, it is necessary to make pharmaceutical studies designed for those in the greatest need of advanced care. Other types of clinical studies must be developed and carried out in those that do not fit into simple double-blind studies. The patients must also be given appropriate attention in every-day care settings.

Due to the complexity of the causes behind MEs and the large differences between work-places, no single action will solve the problems involved and each workplace must work with the safety issues. Work-place routines must pay regards to human weaknesses. Policies and procedures should be easily available, updated, well known and easy to read. The rooms for storage and preparation of medication must be well structured and nurses left undistracted during the medication process. The individual nurse should be trained to make priority to safety rather than being by tradition available to everyone all the time. Nurse leaders must ensure that newly graduated nurses as well as experienced nurses have the necessary skills for their tasks. Workload and the complexity of the tasks must be individualized based on the nurse’s experience and knowledge. Continuous education is crucial for safety in the rapidly changing world of care. An organizational culture that encourages recognition and learning from errors rather than blaming and shaming promotes safety.

Although this was a study analyzing hazardous MEs, 80 % of the patients were not harmed at all. This shows that there is vigilance and attention when errors have occurred in the health care system. An important gate-keeper for MEs is the alert and well informed patient, but the situation is more complicated for the critically ill, very young or very old patient who has to rely on the professional work of the staff. A suggestion for further research is a study of factors that hold back serious consequences of MEs**.**

## Conclusions

To err is human, and the MEs regarded as malpractice in Sweden were of the same character as MEs worldwide. A complex interplay between individual and system factors often contributed to the errors. Workplaces must work systematically with patient safety routines, and care for the well-being and the continuous education of the employees.

## Abbreviation

ICF: Individual contributory factor
IMSN: International medication safety network
IVO: Health and social care inspectorate
ME: Medication error
NBHW: National board of health and welfare (in Sweden)
RN: Registered nurse
SCF: System contributory factor
